# AMON: annotation of metabolite origins via networks to integrate microbiome and metabolome data

**DOI:** 10.1186/s12859-019-3176-8

**Published:** 2019-11-28

**Authors:** M. Shaffer, K. Thurimella, K. Quinn, K. Doenges, X. Zhang, S. Bokatzian, N. Reisdorph, C. A. Lozupone

**Affiliations:** 1 Department of Medicine, University of Colorado Anschutz Medical Campus, Aurora, CO 80045 USA; 2 Skaggs School of Pharmacy and Pharmaceutical Sciences, University of Colorado Anschutz Medical Campus, 80045CO, Aurora, USA; 3 Present address: BioElectron Technology Corporation, Mountain View, CA 94043 USA

**Keywords:** Microbiome, Metabolome, Data-integration

## Abstract

**Background:**

Untargeted metabolomics of host-associated samples has yielded insights into mechanisms by which microbes modulate health. However, data interpretation is challenged by the complexity of origins of the small molecules measured, which can come from the host, microbes that live within the host, or from other exposures such as diet or the environment.

**Results:**

We address this challenge through development of AMON: Annotation of Metabolite Origins via Networks. AMON is an open-source bioinformatics application that can be used to annotate which compounds in the metabolome could have been produced by bacteria present or the host, to evaluate pathway enrichment of host verses microbial metabolites, and to visualize which compounds may have been produced by host versus microbial enzymes in KEGG pathway maps.

**Conclusions:**

AMON empowers researchers to predict origins of metabolites via genomic information and to visualize potential host:microbe interplay. Additionally, the evaluation of enrichment of pathway metabolites of host versus microbial origin gives insight into the metabolic functionality that a microbial community adds to a host:microbe system. Through integrated analysis of microbiome and metabolome data, mechanistic relationships between microbial communities and host phenotypes can be better understood.

## Background

The host-associated microbiome can influence many aspects of human health and disease through its metabolic activity. Examples include host:microbe co-metabolism of dietary choline/carnitine to Trimethylamine N-oxide (TMAO) as a driver of heart disease [1], microbial production of branched chain amino acids as a contributor to insulin resistance [2], and microbial production of 12,13-DiHOME as a driver of CD4^+^ T cell dysfunction associated with childhood atopy [3]. A key way of exploring which compounds might mediate relationships between microbial activity and host disease is untargeted metabolomics (e.g. mass spectrometry) of host materials such as stool, plasma, urine, or tissues. These analyses result in the detection and relative quantitation of hundreds to thousands of compounds, the sum of which is referred to as a “metabolome”. Host-associated metabolomes represent a complex milieu of compounds that can have different origins, including the diet of the host organism and a variety of environmental exposures such as pollutants. In addition, the metabolome contains metabolic products of these compounds, i.e. metabolites, that can result from host and/or microbiome metabolism or co-metabolism [4].

One way to estimate which metabolites in host samples originate from host versus microbial metabolism is to use metabolic networks described in databases such as the Kyoto Encyclopedia of Genes and Genomes (KEGG) [5]. These networks capture the relationship between metabolites, the enzymes that produce them, and the genomes of organisms (both host and microbial) that contain genes encoding those enzymes. These networks thus provide a framework for relating the genes present in the host and colonizing bacteria, and the metabolites present in a sample. Several papers have explored use of metabolic networks to understand likely products of microbial metabolism [6–14]. Algorithms that consider the combined influence of microbial and host metabolism have also been explored [2, 8, 10–12, 15]. Although these studies together show great promise in this field, these methods often rely on comprehensive, validated metabolic models [6, 8, 13, 14], focus only on subsets of carefully measured metabolites [15], or focus on other aspects of community ecology such as predicting metabolic interactions [11], limiting their application to relating complex untargeted metagenomics and metabolomic datasets [16]. Furthermore, algorithms developed in this field often do not have a user interface allowing researchers to apply them to their own data [2, 15, 17]. One exception is the predicted relative metabolic turnover (PRMT) scoring metric [16, 18], and MIMOSA [6], an application that uses PRMT to relate metabolite levels and predicted microbial metabolic capabilities in untargeted metabolomes and metagenomes. However, MIMOSA does not currently evaluate contributions of host metabolism to metabolite levels.

Here we present a tool for annotation of metabolite origins via networks (AMON), which uses information in KEGG to predict whether measured metabolites are likely to originate from singular organisms or collections of organisms based on a list of the genes that they encode. As an example, AMON can be used to predict whether metabolites may originate from the host versus from host-associated microbiomes as assessed with 16S ribosomal RNA (rRNA) gene sequences or shotgun metagenomics. We demonstrate our tool by applying it to a dataset from a cohort of HIV positive individuals and controls in which the stool microbiome was assessed with 16S rRNA gene sequencing and the plasma metabolome was assessed with untargeted liquid chromatography mass spectrometry (LC/MS). We also illustrate how much information is lost when we only focus on compounds and genes of known identity/function, emphasizing the need for complimentary approaches to general metabolomic database searches for the identification of microbially produced compounds.

## Methods

### AMON implementation

**Fig. 1:**
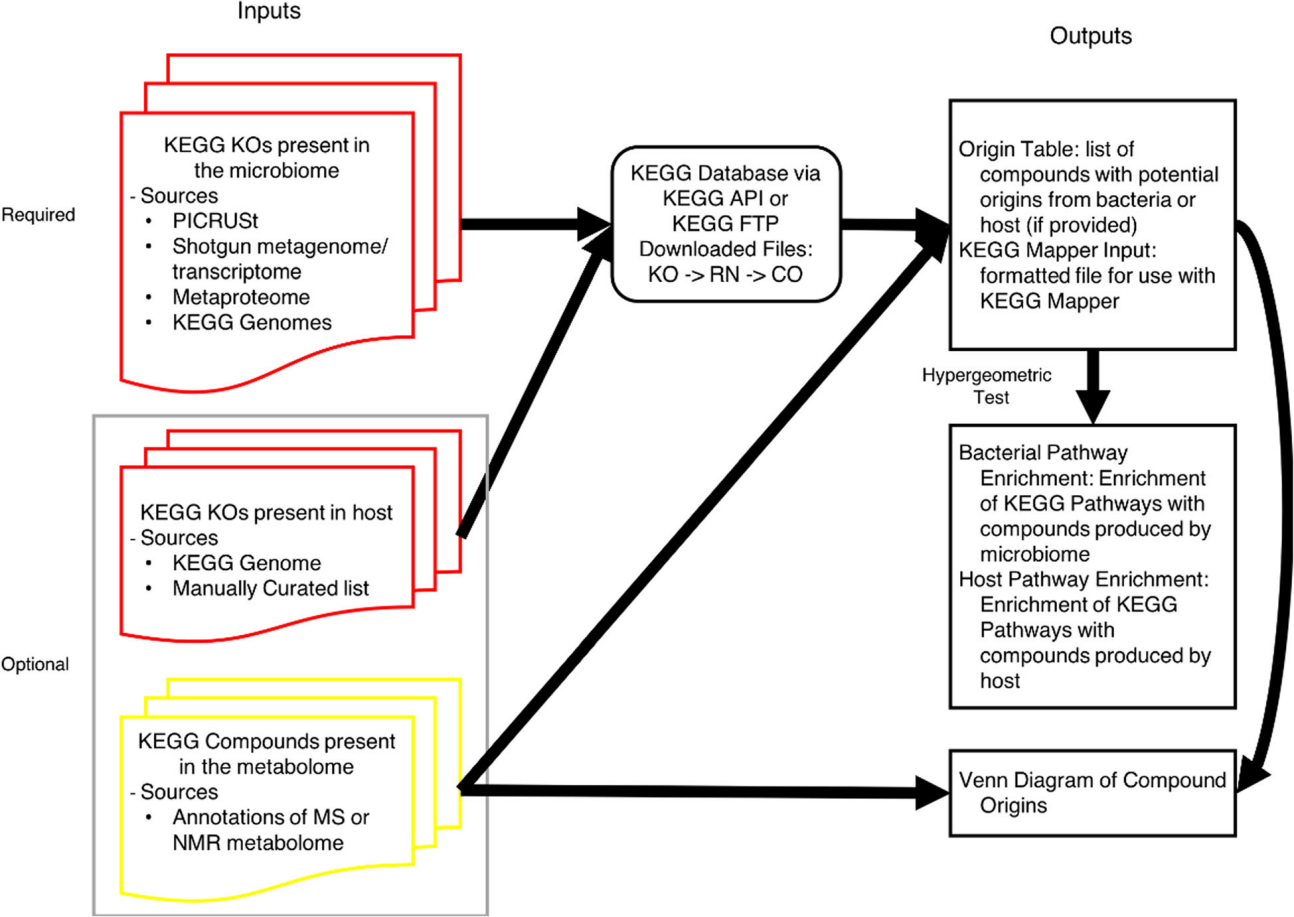
The data flow of AMON. This schematic shows the flow of data through the AMON tool. The required input is a list of KEGG orthology (KO) identifiers which will be used with the KEGG database to determine the possible metabolites produced. This information is output to the user along with a pathway enrichment analysis to show functionality in the produced metabolite and a KEGG mapper file for visualization of metabolite origin in KEGG pathways

AMON is an open source program implemented in python 3. It is available at https://github.com/lozuponelab/AMON as well as in the python package index. AMON takes as input lists of KO (KEGG Orthology) identifiers that are predicted to be present in different potential sources (e.g. the metagenome of a host-associated microbiome or the genome of host organism) and a list of KEGG compound IDs, such as from an annotated metabolome (Fig. 1). Microbiome KO lists can be generated from 16S rRNA data using PICRUSt [19] or Tax4Fun [20], or from a shotgun metagenome using annotation tools such as HUMAnN [21]. The KOs from any KEGG organism can be acquired using the extract_ko_genome_from_organism.py script supplied with AMON**,** which determines the KOs for a given organism from files retrieved using the freely available KEGG API (https://www.kegg.jp/kegg/rest/) or from a user-supplied KEGG FTP file for those with a KEGG subscription.

The goal of AMON is to determine the compounds that a set of KEGG KOs can potentially generate. First, the reactions associated with each KO and formulas describing substrates and products of each reaction are retrieved from the KEGG “reactions” file or the KEGG API. The products of all reactions are the putative set of compounds that the given KOs could produce. The KEGG reaction file does not directly define reversibility of reactions so AMON assumes that the primary direction of reactions is from the left to the right in the equations and therefore the compounds on the right side of the equation are the products. As an example, if the supplied set of KOs included K00929 (butyrate kinase), the following formula from the reaction performed by this enzyme (R01688) would be retrieved: C02527 (Butanoyl phosphate) = > C00246 (butyrate). Butyrate would then be added to the list of compounds that could be generated by this set of KOs.

**Fig. 2:**
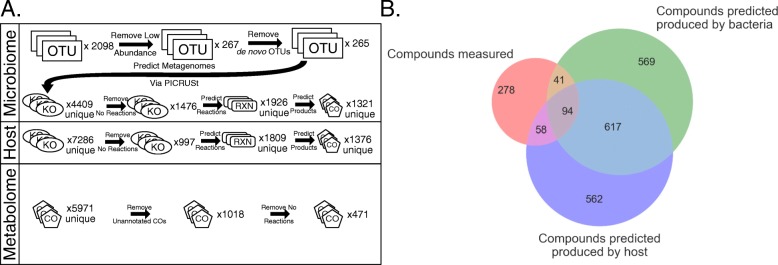
The results of a case study running AMON with 16S rRNA sequencing data from stool and PICRUSt to predict the metagenome along with the KEGG human genome and an LC/MS untargeted metabolome. **a** A flow diagram showing how much data is lost between parts of analyses at all data levels. **b** A Venn diagram showing overlaps in compound sets. The red circle shows compounds detected with untargeted LC/MS with an annotated KEGG compound ID. The green and purple circles show compounds that the metabolic network tells us could have been produced by the bacteria present in the microbiome and the host respectively

**Fig. 3:**
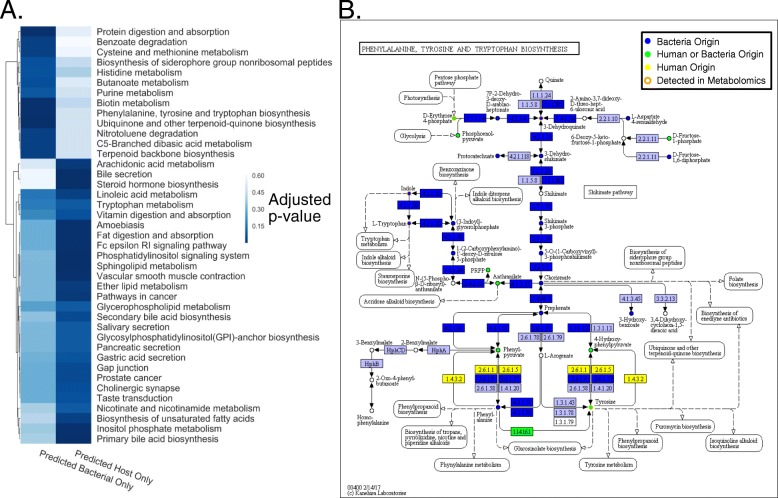
Enrichment of pathways and a single enriched pathway colored with metabolite origin. **a** A heatmap showing the *p*-values associated with a pathway enrichment analysis with KEGG pathways. The first column is p-values for enrichment of KEGG pathways in compounds that were detected via untargeted LC/MS of plasma and we predict could be generated by members of the fecal microbiome. The second column is the same but for compounds that we predicted could have been generated by the human host. **b** This pathway map is colored by putative origin of the compound, which are circles, and presence of the reaction, which are rectangles. Dark blue is a compound or gene with a bacterial origin, yellow is a compound or gene with a human origin, orange outlined compounds are detected in the metabolomics. Circles or rectangles could be of human or bacterial origin

AMON produces a table indicating which compounds could be produced by each of the provided KO sets or both. For instance if one KO set is from the host and one from the microbiome, AMON will indicate whether compounds that were the products of the reactions that these compounds encoded originated from the microbiome KO set only, host KO set only, or both microbial and host KO sets. A file for input to KEGG mapper (https://www.genome.jp/kegg/mapper.html) is also produced, which can be used to overlay this information on KEGG pathway diagrams. AMON also generates information on pathway enrichment in the compounds produced by the user-supplied gene lists. Specifically, the pathway assignment of the set of metabolites predicted to be produced by each input KO list is tested for enrichment relative to the full set of all compounds in that pathway using the hypergeometric test. This calculation is performed for all KEGG pathways that had at least one metabolite predicted to be produced by the provided gene sets. Both raw and Benjamini-Hochberg FDR adjusted *p*-values are reported. AMON also produces a summary figure (Venn diagram) illustrating predicted metabolite origins. A set of example outputs are provided with the case study (Figs. 2b, 3 and Additional file 2: Table S2, Additional file 3: Table S3). We have found run times to typically be less than 1 min if KEGG files are provided. If KEGG files are not provided then run time is dependent on the length of the provided KO lists since the KEGG API limits the volume of data downloaded in a set period of time.

### Case study

We illustrate the utility of AMON using a data set from the gut microbiome (16S rRNA) and blood metabolome (LC/MS) of HIV positive individuals and controls. The cohort and the fecal 16S rRNA data were previously described as part of a larger study of differences in the fecal microbiome in HIV positive and high risk populations [22]. These 16S rRNA data are paired with metabolome data as a part of a study described at ClinicalTrials.gov (Identifier: NCT02258685). Stool samples from 59 individuals, of which 37 were HIV positive and 22 were HIV negative, were collected at home in a commode specimen collector within 24 h of the clinic visit during which blood was drawn.

### Generation of fecal 16S rRNA data

Stool samples were stored at − 20 °C during transit and at − 80 °C prior to DNA extraction with the MoBIO kit and preparation for barcoding sequencing using the Earth Microbiome Project protocol [23]. The 16S rRNA gene V4 region of stool microbes was sequenced using MiSeq (Illumina), denoised using DADA2 [24] and binned into 99% Operational Taxonomic Units (OTUs) using UCLUST [25] and the greengenes database (version 13_8) via QIIME 1.9.1 [26]. We used PICRUSt [19] to predict a metagenome and AMON to predict metabolites.

### Plasma sample preparation

A modified liquid-liquid extraction protocol was used to extract hydrophobic and hydrophilic compounds from the plasma samples [27]. Briefly, 100 μL of plasma spiked with internal standards underwent a protein crash with 400 μL ice cold methanol. The supernatant was dried under nitrogen and methyl *tert*-butyl ether (MTBE) and water were added to extract the hydrophobic and hydrophilic compounds, respectively. The upper hydrophobic layer was transferred to a new tube and the lower hydrophilic layer was re-extracted with MTBE. The upper hydrophobic layer was combined, dried under nitrogen and reconstituted in 200 μL of methanol. The hydrophilic layer was dried under nitrogen, underwent a second protein crash with water and ice-cold methanol (1:4 water-methanol). The supernatant was removed, dried by SpeedVac at 45 °C and reconstituted in 100 μL of 5% acetonitrile in water. Both fractions were stored at − 80 °C until LCMS analysis.

### Liquid chromatography mass spectrometry

The hydrophobic fractions were analyzed using reverse phase chromatography on an Agilent Technologies (Santa Clara, CA) 1290 ultra-high precision liquid chromatography (UHPLC) system on an Agilent Zorbax Rapid Resolution HD SB-C18, 1.8um (2.1 × 100 mm) analytical column with an Agilent Zorbax SB-C18, 1.8 μm (2.1 × 5 mm) guard column. The hydrophilic fractions were analyzed using hydrophilic interaction liquid chromatography (HILIC) on a 1290 UHPLC system using a Phenomenex Kinetex HILIC, 2.6um (2.1 × 50 mm) analytical column with an Agilent Zorbax Eclipse Plus C8 5μm (2.1 × 12.5 mm) guard column. The hydrophobic and hydrophilic fractions were run on Agilent Technologies (Santa Clara, CA) 6520 and 6550 Quadrupole Time of Flight (QTOF) mass spectrometers, respectively. Both fractions were run in positive and negative electrospray ionization (ESI) modes, as previously described [28].

### Mass spectrometry data processing

Compound data was extracted using Agilent Technologies (Santa Clara, CA) Mass Hunter Profinder Version B.08 (Profinder) software in combination with Agilent Technologies Mass Profiler Professional Version 14 (MPP) as described previously [28]. Specifically, a Profinder recursive workflow was used to extract compound data from all samples based on abundance profiles in m/z and retention time (RT) dimensions. The *aqueous positive* mode samples were extracted as follows: RT extraction range 0–14.7 min with noise peak height filter ≥2000 counts, ion species: +H, +Na, +K, +NH4 and charge state maximum of 2. Alignment tolerance for RT was 0% + 0.3 min with mass 20 ppm + 3 mDa. The ‘Find by Molecule Feature’ (MFE) parameters used were height ≥ 4500 counts and a score of 90. The ‘Find by Ion’ (FbI) parameters were height ≥ 3500 for EIC peak integration with post-processing filters using Abs height ≥ 3500 counts and score 50. The *aqueous negative* mode samples were extracted as follows: RT extraction range 0–14.7 min with noise peak height filter ≥1000 counts, ion species: -H, +Cl, +HCOO, +CH3COO and charge state maximum of 2. Alignment tolerance for RT was 0% + 0.3 min with mass 20 ppm + 3 mDa. The MFE parameters used were height ≥ 3000 counts and a score of 90. The FbI parameters were height ≥ 2500 for EIC peak integration with post-processing filters using Abs height ≥ 2500 counts and score 50. The *lipid positive* mode samples were extracted as follows: RT extraction range 0–10.4 min with noise peak height filter ≥500 counts, ion species: +H, +Na, +K, +NH4 and charge state maximum of 2. Alignment tolerance for RT was 0% + 0.25 min with mass 20 ppm + 2 mDa. The MFE parameters used were height ≥ 2000 counts and a score of 90. The FbI parameters were height ≥ 1500 for EIC peak integration with post-processing filters using Abs height ≥ 1500 counts and score 50. The *lipid negative* mode samples were extracted as follows: RT extraction range 0–10.4 min with noise peak height filter ≥300 counts, ion species: -H, +Cl, +HCOO, +CH3COO and charge state maximum of 2. Alignment tolerance for RT was 0% + 0.3 min with mass 20 ppm + 3 mDa. The MFE parameters used were height ≥ 4500 counts, and score 90. The FbI parameters were height ≥ 3500 for EIC peak integration with post-processing filters using Abs height ≥ 3500 counts and score 50. In all cases we required compounds had to be present in at least 2 sample files. Extracted data was imported into MPP and the KEGG database was used to putatively annotate plasma compounds based on exact mass, isotope ratios and isotopic distribution with a mass error cutoff of 10 ppm, whereby the predicted isotope distribution is compared to actual ion height and a score is generated. This corresponds to a Metabolomics Standards Initiative metabolite identification level 3 [29] and a Schymanski identification level 5 [31]. Although our approach in some cases output multiple KEGG compounds as possible “hits,” we selected the compound with the highest score [29] such that each compound was assigned a single KEGG compound ID.

## Results

We used AMON to relate the stool microbiome (as assessed with 16S rRNA gene sequencing) to the plasma metabolome (as assessed with untargeted LC/MS), in a cohort of HIV positive individuals and HIV-negative controls. The overall goal of our case study was to use AMON to determine the degree to which annotated compounds in the plasma metabolome of our study cohort may have been produced by bacteria present in fecal samples, the host, either (i.e. both are capable of production), or neither (i.e. neither the human or the fecal microbiome are predicted to be capable of producing the observed metabolite).

We used the 16S rRNA data and PICRUSt to predict the genome content of the OTUs detected in the fecal samples. PICRUSt drops OTUs from the analysis that do not have related reference sequences in the database and produces an estimate of the nearest sequenced taxon index (NSTI) which measures how close those sequences are to sequenced genomes (those more closely related to genomes have more power to make predictions regarding gene content). Since human gut bacteria are well represented in genome databases, only 0.7% of total reads of the detected sequences were dropped on account of not having a related reference sequence in the database. Furthermore, the average NSTI across samples was 0.08, indicating that most OTUs were highly related to an organism with a sequenced genome. We applied PICRUSt to the 16S rRNA dataset with only OTUs present in more than 11 of 59 samples (20%) included. The 267 remaining OTUs were predicted to contain 4409 unique KOs using PICRUSt. We used the KEGG list of KOs in the human genome to represent human gene content.

We provided these lists of gut microbiome and human KOs to AMON to produce a list of compounds generated from the gut microbiome and the human genome. We also provided AMON with a reaction file downloaded from KEGG January of 2015. Of the 4409 unique KOs that PICRUSt predicted to be present in the gut microbiome, only 1476 (33.5%) had an associated reaction in KEGG. Those without associated reactions may represent orthologous gene groups that do not perform metabolic reactions (such as transporters), or that are known to exist but for which the exact reaction is unknown, showing gaps in our knowledge (Fig. 2a). Using information in KEGG, AMON predicted these KOs to produce 1321 unique compounds via 1926 unique reactions. The human genome was predicted to produce 1376 metabolites via 1809 reactions.

Our metabolomics assays detected 5971 compounds, of which only 1018 (17%) could be putatively annotated with KEGG compound identifiers via a database search and based on match of measured m/z to KEGG compound mass within 10 ppm. Further, only 471 (6%) of the 5971 detected compounds were associated with a reaction in KEGG (Additional file 1: Table S1). Of these 471 annotated compounds in the plasma metabolome with associated KEGG reactions, 189 were predicted to be produced by enzymes in either human or stool bacterial genomes as follows: 40 compounds were exclusively produced by bacteria, 58 exclusively by the host, and 91 by either human or bacterial enzymes (Fig. 2b; Additional file 2: Table S2). There were a remaining 282 compounds that had KEGG compound IDs associated with at least one reaction but were not predicted to be from the human or the gut microbiome. These may be 1) from the environment, 2) produced by microbes in other body sites, 3) host or gut microbial products from unannotated genes, 4) artifacts derived from metabolite decompositions in the samples and/or are mis-annotations via the matching based on m/z alone.

We used AMON to assess enrichment of pathways in the detected human and bacterial metabolites using the hypergeometric test (Fig. 3a; Additional file 3: Table S3). The 40 compounds predicted to be produced by stool bacteria and not the host were enriched in xenobiotic degradation pathways, including nitrotoluene and atrazine degradation, and pathways for amino acids metabolism, including the phenylalanine, tyrosine and tryptophan biosynthesis pathway and the cysteine and methionine metabolism pathway. The metabolite origin data was visualized using KEGG mapper for the phenylalanine, tyrosine and tryptophan biosynthesis pathway (Fig. 3b). This tool helps to visualize the host-microbe co-metabolism and which genes are important for compounds that may have come from multiple sources. For instance, Fig. 3b allows us to see that indole is a compound found in our metabolome that could only have been produced by bacterial metabolism via the highlighted enzyme (K01695, tryptophan synthase). Also, tyrosine is a compound found in our metabolome that could have been synthesized by a variety of enzymes found only in bacteria, only in humans, or in both and so further exploration would be needed to understand origins of this compound. The 58 compounds which were detected and predicted to be produced by the human genome were enriched in pathways that include bile secretion, steroid hormone biosynthesis and gastric acid secretion.

### Comparison of AMON with MIMOSA

The functionality of AMON is related to that of another tool called MIMOSA [6], in that MIMOSA also uses PICRUSt and KEGG to integrate microbiome (16S rRNA) and metabolome data. Unlike AMON, MIMOSA does not relate contributions of microbial versus host metabolism. However, MIMOSA determines quantitative relationships between the relative abundance of genes in a metagenome and the abundance of the particular compounds in a metabolome that their gene products produce/degrade. To compare the results of AMON and MIMOSA when applied to the same dataset, we analyzed our HIV case study with MIMOSA (Additional file 4: Table S4). We supplied MIMOSA with 1) a table of compound abundances measured in our HIV samples with untargeted LC/MS, 2) a gene abundance and gene contributions file generated using 16S rRNA data and PICRUSt and 3) a reaction_mapformula.lst file downloaded from KEGG in January 2015. Of 1018 compounds with KEGG annotations, MIMOSA was able to successfully analyze the potential microbe contributions for 57 different compounds, and of these 10 (17.5%) had significant correlations to metabolic potential scores and were thus considered “well-predicted”. In contrast, AMON predicted 135 compounds in the plasma metabolome to have derived either exclusively from the microbiome (*n* = 40) or from the microbiome or host (*n* = 91). Metabolites that AMON predicted to be of exclusive microbial (but not host) origin that MIMOSA was unable to analyze included important microbially-produced signaling molecules such as indole [32, 33], butyrate [34], D-alanine [35], and known microbial metabolites of dietary components such as 4-hydroxybenzoic acid [36] and diacetyl [37].

Of the 57 metabolites analyzed by MIMOSA, only 22 were predicted to be of bacterial origin by AMON. Some compounds analyzed by MIMOSA that were not predicted by AMON to be of microbial origin were substrates and not products in microbial reactions. This reflects the different goals of the programs to predict metabolite origins (AMON) versus metabolite turnover that may be influenced by production or degradation (MIMOSA). Three compounds that AMON determined that the host and the microbiome could produce were well-predicted by MIMOSA. These included biliverdin (C00500) and cell membrane components phosphatidylethanolamine (C00350) and 1-Acyl-sn-glycero-3-phosphocholine (C04230).

## Discussion

Taken together, these analyses show that AMON can be used to predict the putative origin of compounds detected in a complex metabolome. Our case study shows the specific application of predicting origins of plasma compounds as being from the fecal microbiome versus the host. However, this tool can be used to compare any number of different sources – e.g. from the microbiomes of different body sites or compounds that may come directly from plants consumed in the diet. Also, the outputs of AMON can be used in conjunction with lists of metabolites that were determined to significantly differ with disease state or correlate with other host phenotypes to predict origins of metabolites of interest.

AMON uses the latest updates of KEGG while not requiring the user to purchase a KEGG license, by using either user supplied files for those with a license or the KEGG API which is freely available. However, we do note that the KEGG API option is comparatively slow and limits the maximum dataset size (due to limits of the KEGG API). AMON is built to be flexible to the methods used to obtain the list of KOs present in each source sample and compounds present in a metabolome. Although our example uses PICRUSt to predict compounds of bacterial origin using 16S rRNA sequence data, AMON requires a list of KEGG Orthology identifiers as input and so could also be used with shotgun sequencing data. This can allow for a more thorough interrogation of host microbiomes that account for strain level variation in genome content and opens its application to environments with less understood genomes.

The pathway enrichment of compounds predicted to be unique to the gut microbiome and the host provide a level of validation for AMON results. The pathways enriched with compounds predicted to only be from microbes are consistent with known roles for gut bacteria in degrading various xenobiotics [38–42] and for influencing amino acid [43, 44] and vitamin metabolism [45]. Likewise, the pathways enriched with compounds predicted to be human only include host processes such as taste transduction and bile secretion. Further, since the microbial community measured was from the human gut and the metabolome from plasma, these results suggest that these may represent microbial metabolites that have translocated from the gut into systemic circulation, although validation of the identity of these compounds with authentic standards would be needed to confirm these results. Several studies that have shown a strong influence of the gut microbiome on the plasma metabolome (reviewed in [4]) and the gut microbiome has been linked with many diseases that occur outside of the gut. Examples include interactions between the gut and brain via microbially derived compounds such as serotonin [44], and branched chain amino acids from the gut microbiome as a contributor to insulin resistance [2].

The most similar tool to AMON is MIMOSA [6]. While AMON’s goal is to predict whether a compound could have been produced by community of bacteria versus the host, MIMOSA is a relatively quantitative tool that produces information on which particular microbes may influence which particular microbial metabolite levels, and considers both productive and consumptive relationships in these calculations. Unlike AMON, MIMOSA does not incorporate knowledge of host metabolism.

AMON designated many more compounds in the plasma metabolome of being of potential microbial origin compared to MIMOSA when run on the same dataset, and these included important microbially-produced signaling molecules such as indole [32, 33]. One potential reason for this may be more strict criteria needed for forming a metabolic potential score in MIMOSA, as they note in their paper that roughly 50% of metabolites in each data set could not be scored [6]. However, another source of this difference may be the KEGG source file used to define reactions. AMON uses the “reaction” file provided by KEGG which details all reactions in the KEGG database and MIMOSA uses the “reaction_mapformula.lst” file, which also gives pathway specific information for each reaction (although MIMOSA does not currently use this additional information). We chose to use the “reaction” file of KEGG because it contains information for more reactions than the reaction_mapformula.lst file (e.g. 11,196 versus 7420 for files downloaded on June 9, 2019). The PRMT algorithm used by MIMOSA also makes many assumptions to perform a quantitative analysis that AMON does not, including that that relative abundance of genes for a unique enzyme function reflects levels of expressed functional proteins and reaction rates. Although the PRMT algorithm generally and MIMOSA specifically have been shown to provide strong correlations between microbiome functionality and metabolites and biological insights [6, 17], these weaknesses indicate that the broader information of microbe produced metabolites that is not reliant on this quantitative information that AMON produces is also valuable.

However, for compounds that were evaluated by both MIMOSA and AMON, using the two tools together provided interesting and complimentary insights. In particular, 3 compounds that AMON determined that both the host and the microbiome could produce were well-predicted by MIMOSA, supporting that gut microbe metabolism is an important driver of levels of these compounds despite the ability of the host to produce them. One of these is biliverdin, which is produced by macrophages during heme catabolism but also produced by heme oxygenenases encoded by a variety of bacteria that utilize heme as a source of iron [46]. The other two were lipids that are common components of bacterial cell membranes, supporting that cellular components of bacteria shape the plasma metabolome.

Our analysis also highlights limitations of these approaches that use functional databases such as KEGG due to issues with annotation of both metabolites and the enzymes that may produce them. Overall, it is striking that of 5971 compounds in the LC/MS data, only 471 could be linked to enzymatic reactions in KEGG. For example the human genome is known to contain approximately 20,000 genes [47]; however, there are only 7286 KOs annotated in KEGG. These KOs only predict the creation of 1376 unique compounds while the Human Metabolome Database 4.0 contains 114,100 [48]. Part of this discrepancy is because multiple species of lipids are, generally, reduced to a single compound in KEGG. For example, while KEGG includes a single phosphatidylcholine (PC) lipid molecule in the glycerophospholipid pathway, in fact, there are over 1000 species of PCs. It is also important to note that metabolite annotations are based on peak masses and isotope ratios, which can often represent multiple compounds and/or in-source fragments; our confidence in the identity of these compounds is only moderate. As with any metabolomics dataset, we caution the user to limit their biological conclusions when level 3 annotations are used in downstream applications such as AMON. As it is not feasible to verify compound identities using authentic standards or MS/MS for hundreds of compounds, AMON provides a valuable tool for prioritizing compounds for additional analysis, including identification using authentic standards, by providing information on their potential origins.

The limitations are more stark for complex microbial communities, where there are fewer genes of known function. Because of these gaps in our knowledge of metabolite production, efforts to identify microbially produced metabolites that affect disease should also use methods that are agnostic to these knowledge-bases. These include techniques such as 1) identifying highly correlated microbes and metabolites to identify potential productive/consumptive relationships that can be further validated 2) molecular networking approaches which take advantage of tandem mass spectroscopy data to annotate compounds based on similarity to known compounds with related tandem mass spectrometry (MS/MS) profiles [49] or 3) coupling LC/MS runs with data from germ-free versus colonized animals [1, 50, 51] or antibiotic versus non-antibiotic treated humans [52, 53]. Because AMON takes only KO identifiers and can pull database information from the KEGG API or user provided KEGG files, our tool will become increasingly useful with improvements from KEGG as well as other parts of the annotation process. In addition, AMON can also accept metabolomics datasets with Level 1 identifications; i.e. where the identity of the compounds has been verified with authentic standards.

Although our application is specifically designed to work with the KEGG database, similar logic could be used for other databases such as MetaCyc [54]. Our tool also does not apply methods such as gap-filling [7, 55] and metabolic modeling [12, 57] in its estimates. The goal is not to produce precise measurements of the contributions of the microbiome and host to the abundance of a metabolite. Rather, AMON is designed to annotate metabolomics results to give the user an understanding of whether specific metabolites could have been produced directly by the host or microbial communities. If a metabolite is identified by AMON as being of microbial origin and is associated with a phenotype, this result should motivate the researcher to perform follow up studies. These can include confirming the identity of the metabolite, via methods such as tandem mass spectrometry, and performing experiments to confirm the ability of microbes of interest to produce the metabolite.

AMON also does not account for co-metabolism between the host and microbes. An example of this is the production of TMAO from dietary choline. Our tool would list TMAO as a host compound and its precursor trimethylamine (TMA) as a microbiome derived compound but would not indicate that TMAO could overall not be produced from dietary substrates unless a microbiome was present. Further inspection of metabolic networks, which is enabled by AMON’s functionality in producing outputs for visualization in KEGG mapper may be needed to decipher these co-metabolism relationships. Previously described methods for constructing possible biotransformation pathways, while discriminating between microbiota and host reactions [15] could also be incorporated into AMON in the future.

## Conclusions

When researchers are seeking to integrate microbiome and metabolome data, identifying the origin of metabolites measured is an obvious route. AMON facilitates the annotation of metabolomics data by tagging compounds with their potential origin, either as bacteria or host. This allows researchers to develop hypotheses about the metabolic involvement of microbes in disease.

## Supplementary information

**Additional file 1: Table S1.** Table of Metabolites with KEGG Annotations from LC/MS of Human Plasma

**Additional file 2: Table S2**. Example Output of AMON Metabolite Origin Table

**Additional file 3: Table S3.** Example Output of AMON Pathway Enrichment Table

**Additional file 4: Table S4.** MIMOSA Results Table from Case Study Data

## Data Availability

Microbiome data is available in the European Nucleotide Archive repository **PRJEB28485** (https://www.ebi.ac.uk/ena/data/view/PRJEB28485). The metabolomics data is available at the NIH Common Fund’s National Metabolomics Data Repository (NMDR) website, the Metabolomics Workbench, https://www.metabolomicsworkbench.org, where it has been assigned Project ID **(ST001268)**. The data can be accessed directly via its Project DOI:**(**10.21228/M8F108**)**. The genes predicted to be present in this data set and the compounds detected in the metabolomics data are listed in the AMON repository (https://github.com/lozuponelab/AMON/tree/master/data).

## Abbreviations

AMON: Analysis of Metabolite Origins Using Networks
HILIC: Hydrophilic interaction liquid chromatography
KEGG: Kyoto Encyclopedia of Genes and Genomes
KO: KEGG Orthology
LC/MS: Liquid Chromatography / Mass Spectrometry
MPP: Mass Profiler Professional
MS/MS: Tandem mass spectrometry
MTBE: Methyl tert-butyl ether
OTU: Operational Taxonomic Unit
PRMT: Predicted Relative Metabolic Turnover
QTOF: Quadrupole Time of Flight
rRNA: ribosomal RNA
RT: Retention Time
TMA: Trimethylamine
TMAO: Trimethylamine N-oxide
UHPLC: Ultra-high precision liquid chromatography
